# Reliability-Aware Semantic Gating with Retrieval-Augmented Association for Online Multi-Object Tracking Under Industrial Low-Altitude Proxy Conditions

**DOI:** 10.3390/jimaging12090449

**Published:** 2026-09-16

**Authors:** Rongzuo Guo, Yuhang He

**Affiliations:** School of Computer Science, Sichuan Normal University, Chengdu 610068, China; 20241393048@stu.sicnu.edu.cn

**Keywords:** multi-object tracking (MOT), observation-centric SORT (OC-SORT), contrastive language-image pre-training (CLIP), semantic noise, dynamic semantic gating, retrieval-augmented data association, industrial low-altitude proxy conditions

## Abstract

Industrial low-altitude multi-object tracking is challenged by overhead viewpoints, dense small targets, mutual occlusion, homogeneous appearances, and domain-shifted backgrounds. Vision-language pre-trained models provide useful semantic priors, but their category-level representations can mislead instance-level association when many same-class targets are densely distributed. This study develops an OC-SORT-based framework with reliability-aware semantic gating and retrieval-augmented trajectory memory. On the MOT17 reporting split (MOT17-09/10/11/13) with FRCNN detections, the FullModel achieves 46.41% MOTA, 40.00% IDF1, 34.542% HOTA, 26.686% AssA, and 382 identity switches for seed 42. Relative to fixed-weight CLIP+FAISS, it reduces identity switches and fragmentations while trading 0.84 MOTA points for a 0.68-point IDF1 gain; exact reruns with three seeds reproduce the same MOTA, IDF1, and IDSw totals. The VisDrone-derived subset is used only as an industrial low-altitude proxy stress test: its high precision coexists with very low recall, so the result exposes domain-shift limitations, rather than demonstrating deployment-level industrial performance.

## 1. Introduction

Multi-object tracking (MOT) aims to simultaneously estimate the locations of multiple targets and maintain their identity consistency in consecutive video frames [1,2]. It is a fundamental component of visual perception systems such as intelligent surveillance, industrial inspection, warehouse logistics, and autonomous driving. With the increasing demand for industrial automation and intelligent safety supervision, stable, real-time, and identity-preserving MOT methods in complex scenarios have become highly important. However, in industrially relevant environments such as factory workshops, logistics warehouses, and low-altitude inspection scenes, dense target distributions, long-term occlusions, highly similar appearances, and drastic viewpoint changes often occur simultaneously. These factors make reliable identity association challenging for existing tracking algorithms.

Compared with standard pedestrian surveillance benchmarks, industrial low-altitude scenes exhibit several distinctive challenges. First, overhead or oblique viewpoints make target scales highly unstable and reduce the discriminative details of human bodies, vehicles, and equipment. Second, workers often wear similar uniforms, helmets, or reflective vests, leading to strong appearance homogeneity. Third, industrial facilities such as forklifts, conveyors, pallets, shelves, and safety barriers frequently occlude moving targets and generate structured background interference. Fourth, low-altitude unmanned aerial vehicle (UAV) platforms introduce viewpoint changes and non-stationary observation geometry, making constant-velocity motion prediction less reliable. These factors make industrial MOT different from general pedestrian tracking, where identity association can often rely on relatively stable appearance and motion cues. Therefore, industrial low-altitude MOT requires not only general tracking robustness, but also scene-adaptive semantic priors and reliability-aware cue regulation.

In recent years, tracking-by-detection methods have achieved substantial progress in MOT. Simple Online and Real-time Tracking (SORT) [3], Deep SORT (DeepSORT) [4], Observation-Centric SORT (OC-SORT) [5], ByteTrack [6], StrongSORT [7], and Deep OC-SORT [8] have improved online tracking performance from different perspectives, including motion prediction, appearance representation, utilization of low-confidence detections, and trajectory recovery. Meanwhile, You Only Look Once (YOLO)-series detectors have provided efficient detection front-ends for real-time MOT [9,10,11]. Re-identification (ReID) features, pose alignment, and pairwise similarity optimization have also played important roles in distinguishing occluded targets and visually similar objects [12,13,14,15]. More recently, contrastive language-image pre-training (CLIP) [16] models have been introduced into open-vocabulary detection, cross-domain recognition, and MOT tasks, providing new semantic priors for visual target association [17,18].

However, direct transfer from standard surveillance benchmarks to industrial low-altitude scenes remains difficult. To quantitatively characterize this degradation, a controlled reproduction is conducted on the VisDrone2019-MOT-val industrial proxy subset [19] under the same YOLOv8s detections. Multi-Object Tracking Accuracy (MOTA), Identity F1 Score (IDF1), recall, and precision are used as evaluation metrics. OC-SORT obtains −22.3% MOTA, 7.2% IDF1, 16.4% recall, and 45.2% precision, while ByteTrack obtains −21.8% MOTA, 8.1% IDF1, 17.6% recall, and 46.5% precision. The negative MOTA values indicate that false positives, missed detections, and identity switches (IDSw) dominate the correct tracking outputs, whereas the low IDF1 values reveal severe identity inconsistency. These results show that mainstream trackers suffer from substantial performance degradation under overhead viewpoints, dense small targets, frequent occlusion, and domain-shifted backgrounds.

It should be noted that the VisDrone2019-MOT-val industrial proxy subset is not a complete factory-floor MOT benchmark. It is used in this paper only as a proxy stress test for overhead viewpoints, dense small targets, and complex backgrounds. Therefore, the results should not be interpreted as proof of deployment-level industrial performance; they mainly reveal the failure modes and transfer limitations that must be addressed before real factory-floor application.

Existing studies mainly focus on the open-category generalization ability of vision-language models, while insufficient attention has been paid to the potential negative effects of their category-level semantic features in instance-level association among dense objects of the same class. In industrial scenarios, workers wearing uniform clothing, devices of similar models, and dense spatial layouts are common. If CLIP semantic features are directly fused with fixed weights, category consistency may be enhanced while individual differences are weakened, thereby increasing the risk of IDSw.

To address this issue, this paper investigates the reliability of vision-language semantic features in instance-level MOT association and proposes a reliability-aware semantic gating and retrieval-augmented MOT method. Based on OC-SORT, the proposed method analyzes the applicability boundary of CLIP category-level semantic features from the perspective of trajectory-detection candidate pairs. A semantic noise event is defined as the situation in which the semantic similarity between an incorrect same-class candidate detection and the trajectory prototype is no lower than that between the true matched detection and the trajectory prototype. On this basis, several offline metrics are constructed, including semantic discriminative margin, Semantic Noise Rate (SNR), Semantic Noise Magnitude (SNM), and Semantic Collapse Index (SCI), to characterize the misleading risk of CLIP semantic features in instance-level association.

Furthermore, a candidate-pair-level dynamic semantic gating (DSG) mechanism is designed. It estimates semantic reliability using detection confidence, spatial visibility, motion consistency, and target density, and adaptively regulates the CLIP visual semantic term and dictionary-retrieval term. In addition, retrieval-augmented semantic memory is constructed to enhance semantic stability under occlusion recovery, appearance degradation, and homogeneous target association. In the current implementation, the semantic dictionary is used as an auxiliary retrieval reference, rather than as a complete industrial knowledge base.

The main contributions of this paper are summarized as follows.

(1) Semantic noise quantification for CLIP-assisted MOT. This paper analyzes the negative effect of CLIP category-level semantics in instance-level trajectory association. From the perspective of trajectory-detection candidate pairs, semantic noise events are defined, and semantic discriminative margin, Semantic Noise Rate (SNR), Semantic Noise Magnitude (SNM), and Semantic Collapse Index (SCI) are introduced to quantify the risk of same-class but different-identity targets becoming over-clustered in the CLIP feature space.
(2) Reliability-aware dynamic semantic gating. A candidate-pair-level reliability estimation mechanism is proposed to regulate semantic costs during data association. Detection confidence, spatial visibility, motion consistency, and target density are used as observable reliability cues. The resulting gating coefficient dynamically suppresses CLIP semantic cost and dictionary-retrieval cost under unreliable or highly ambiguous association states.
(3) Retrieval-augmented trajectory semantic memory. A retrieval-augmented semantic memory is constructed to provide temporally smoothed semantic references for trajectory association. Instead of using CLIP features as fixed-weight matching cues, the proposed method treats semantic information as a reliability-constrained auxiliary signal, which improves robustness against semantic noise and supports preliminary transfer to industrial low-altitude proxy scenes.

The remainder of this paper is organized as follows. Section 2 reviews related studies on object detection, multi-object tracking, occlusion and similar-appearance handling, vision-language models in MOT, and industrial low-altitude tracking scenarios. Section 3 presents the proposed retrieval-augmented reliability-aware semantic association model. Section 4 evaluates the proposed method from the perspectives of comparison with mainstream trackers, semantic noise analysis, module ablation, reliability-grouped analysis, cross-scenario transfer, runtime efficiency, and failure-case analysis. Section 5 discusses the results. Section 6 concludes the paper and discusses future research directions.

## 2. Related Work

### 2.1. Object-Detection Algorithms

Object detection is an essential front-end for MOT. Its accuracy, recall, and real-time performance directly affect subsequent trajectory association. In recent years, object detection has evolved from traditional closed-set detection to real-time detection, open-vocabulary detection, and vision-language joint perception. Zou et al. [20] systematically reviewed the development of object detection from handcrafted features and convolutional neural networks to Transformer-based detectors, and pointed out that small objects, occlusion, long-tailed categories, and complex backgrounds remain major factors limiting detection performance. Meanwhile, YOLO-series methods have become widely used detection front-ends in online MOT, due to their favorable speed–accuracy trade-off [9,10,11]. Real-time Transformer detectors such as RT-DETR further indicate that global attention modeling provides new structural choices for real-time detection [21,22].

Although existing detectors have achieved high performance in standard scenarios, industrial scenarios still suffer from missed detections and localization errors caused by occlusion, low illumination, small-scale objects, and overhead viewpoints. Detection errors are further amplified during tracking, leading to trajectory fragmentation, false association, and identity switches. Therefore, improving detector performance alone is insufficient to fully solve tracking instability in complex scenarios. More robust multi-cue fusion and reliability regulation mechanisms are still needed in the data association stage.

### 2.2. Multi-Object Tracking Algorithms

Current mainstream MOT methods are mainly based on the tracking-by-detection paradigm [1,2]. Candidate bounding boxes are first generated by a detector in each frame, and then data association is performed to maintain identity continuity. SORT [3] establishes a simple and efficient online tracking framework using Kalman filtering and Hungarian matching. DeepSORT [4] introduces ReID appearance features to improve the discrimination of visually similar targets. ByteTrack [6] effectively exploits low-confidence detections to improve target recall in occlusion and missed-detection scenarios. OC-SORT [5] improves motion prediction and trajectory association from an observation-centric perspective, enhancing trajectory stability under occlusion. StrongSORT [7] and Deep OC-SORT [8] further integrate appearance modeling, trajectory recovery, and multi-cue association strategies to advance online MOT performance.

Other representative tracking-by-detection methods, such as BoT-SORT [23], FairMOT [24], Tracktor [25], and CenterTrack [26], also improve MOT from the perspectives of robust association, joint detection–re-identification learning, regression-based tracking, and point-based tracking. In addition, Transformer-based and end-to-end tracking methods such as TrackFormer [27], MOTR [28], and MOTRv2 [29] attempt to model temporal association through object queries and integrate detection and association into a unified framework. Dataset studies such as DanceTrack [30] and SportsMOT [31] further demonstrate that in scenarios with highly uniform appearances and complex motion patterns, relying solely on appearance features is insufficient to maintain stable identity association.

Overall, existing methods have achieved important progress in motion modeling and appearance representation, but how to dynamically regulate the reliability of different association cues according to target states remains an open problem in complex industrial scenarios.

### 2.3. Occlusion and Similar-Appearance Handling

Occlusion and similar appearance are key factors affecting identity consistency in MOT. Existing studies on occlusion mainly follow two directions: improving detection robustness and optimizing trajectory association. One class of methods enhances the detector’s perception of occluded targets through data augmentation, occlusion simulation, or network-structure improvement. Another class uses motion prediction, trajectory completion, and re-identification mechanisms to maintain identity continuity after short-term occlusion. These methods alleviate trajectory fragmentation to some extent, but substantial uncertainty remains under long-term full occlusion or dense target interaction.

For distinguishing targets with similar appearances, ReID features are commonly used as identity representation cues. Ye et al. [12] provided a systematic review of deep person re-identification. FastReID [13] offers a comprehensive training and evaluation framework for general instance re-identification. Pose-guided feature alignment [14] and Circle Loss [15] improve feature discriminability from the perspectives of local alignment and pairwise similarity optimization, respectively. However, targets in industrial scenarios often exhibit highly uniform appearances, such as workers wearing similar uniforms, equipment of similar models, and repeatedly appearing safety facilities. In such cases, traditional appearance features are prone to homogenization, and relying solely on ReID or local visual texture is insufficient to distinguish different instances. Therefore, higher-level semantic priors should be introduced, while the reliability of semantic information must be constrained according to target states.

### 2.4. Applications and Challenges of CLIP in MOT

CLIP [16] learns strong cross-modal semantic alignment through large-scale image–text contrastive learning, promoting the development of open-vocabulary detection, open-category recognition, and general visual perception. OpenCLIP [32] and SigLIP [33] further verify the scalability and stability of image–text contrastive learning in large-scale pre-training. ViLD [34], GLIP [35], RegionCLIP [36], Detic [37], and Grounding DINO [38] improve open-vocabulary object perception through knowledge distillation, phrase grounding, region-level image–text pre-training, and text-guided detection. In addition, general visual models such as BLIP-2 [39] and Segment Anything [40] demonstrate that large-scale pre-trained models can provide richer semantic representations for complex vision tasks.

In MOT, vision-language models are mainly used to enhance open-category recognition or cross-domain generalization. OVTrack [17] shows that CLIP semantic features can provide effective complementary information for open-vocabulary MOT. However, CLIP features intrinsically emphasize category-level semantic consistency, whereas identity preservation in MOT requires distinguishing different instances within the same category. When targets are densely distributed and highly similar in appearance, category-level semantic features may pull different individuals too close in the feature space, thereby weakening instance-level discrimination. Existing studies have insufficiently discussed this negative effect, and lack mechanisms to dynamically adjust semantic weights according to detection confidence, occlusion degree, and motion consistency. Therefore, how to exploit vision-language semantic priors while suppressing their misleading effects under low-reliability association states is the core problem addressed in this paper.

### 2.5. Multi-Object Tracking in Industrial and Low-Altitude Scenarios

Industrial-related MOT studies can be roughly divided into low-altitude UAV tracking, traffic-surveillance tracking, and factory or warehouse monitoring. VisDrone [19] and UAVDT [41] focus on UAV-view detection and tracking, where small targets, camera motion, and drastic viewpoint changes are dominant challenges. UA-DETRAC [42] and BDD100K [43] provide large-scale traffic scenarios with vehicle density, illumination changes, and long-term occlusion. Compared with these settings, factory and warehouse scenarios further introduce domain-specific difficulties, including uniform workwear, repeated equipment patterns, safety-facility occlusions, and structured production layouts. These factors make identity association more difficult than in general pedestrian or traffic benchmarks.

Most existing public MOT benchmarks do not fully cover factory-floor production scenes. UAV-view datasets such as VisDrone and UAVDT emphasize small targets, camera motion, and viewpoint changes, while traffic datasets such as UA-DETRAC and BDD100K mainly focus on road vehicles. Factory and warehouse scenarios may additionally contain uniform workwear, repeated equipment layouts, conveyor facilities, pallets, and safety barriers. Due to the limited availability of public factory-floor MOT benchmarks, this paper uses a VisDrone-derived industrial proxy subset only for preliminary cross-scenario transfer analysis. This subset is intended to approximate several visual characteristics of industrial low-altitude scenes, including overhead viewpoints, dense small targets, and complex backgrounds. It should not be regarded as a complete factory-floor MOT benchmark.

## 3. Materials and Methods

### 3.1. Overall Framework

**Figure 1:**
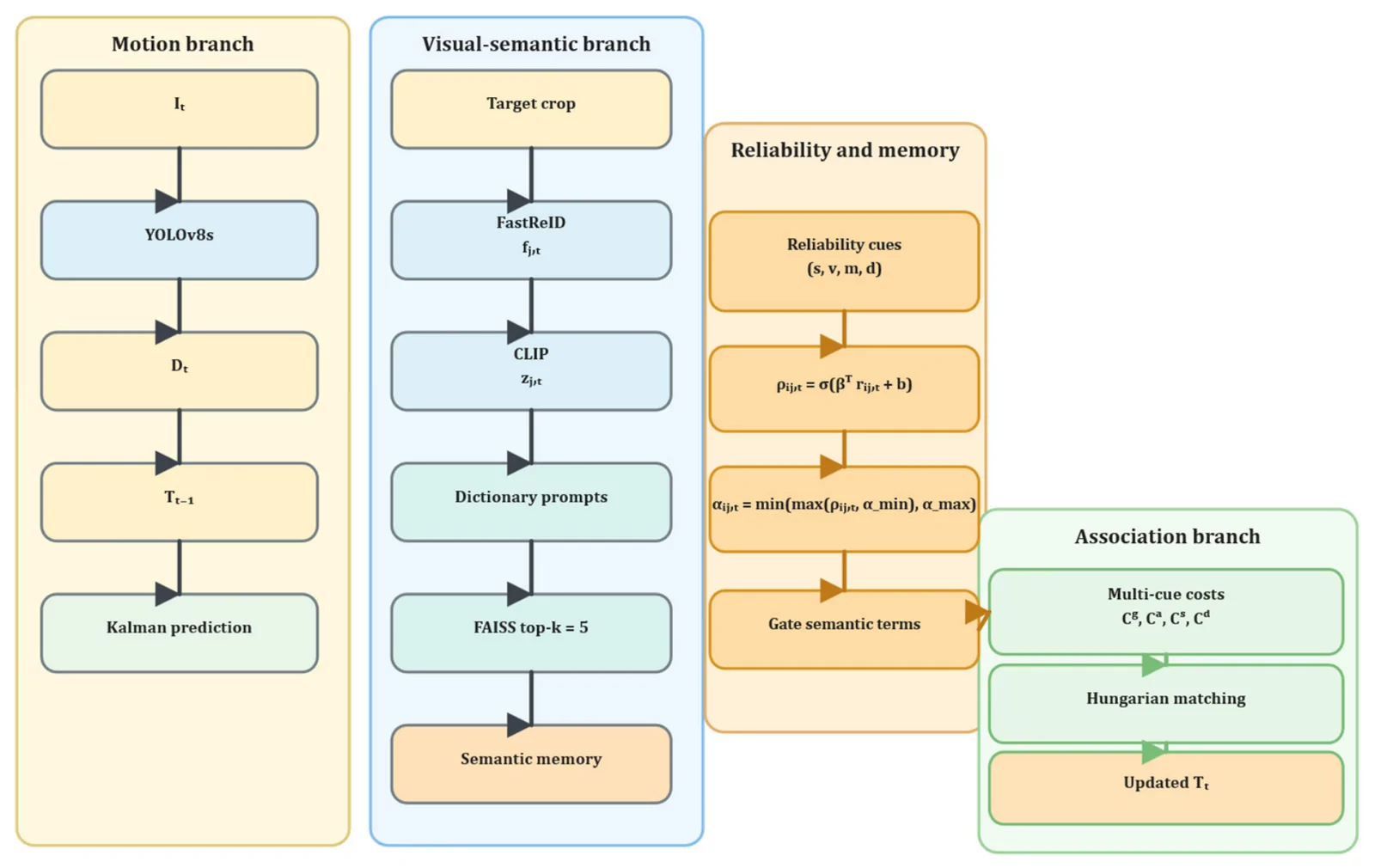
Vector schematic of the proposed reliability-aware semantic gating and retrieval-augmented MOT framework.

To improve identity preservation in complex industrial scenarios, this paper constructs a retrieval-augmented reliability-aware semantic association model based on OC-SORT [5]. The proposed model integrates geometric motion, appearance representation, CLIP visual semantic features, and dictionary-retrieval semantic representation into the data association cost. A candidate-pair-level dynamic gating mechanism is used to regulate the contribution of semantic terms under different association states. The overall framework is shown in Figure 1.

In the proposed framework, the detector first generates candidate observations in the current frame. OC-SORT predicts historical trajectory states using Kalman filtering [44]. For each retained detection crop, FastReID extracts a 2048-dimensional appearance descriptor and CLIP-ViT-B/32 extracts a 512-dimensional L2-normalized visual-semantic feature. The CLIP feature is queried against the text-prompt dictionary through FAISS IndexFlatIP; the top-k = 5 inner-product neighbors are used to form a sparse dictionary response before softmax normalization. For each trajectory-detection candidate pair, the model estimates semantic reliability from detection confidence, spatial visibility, motion consistency, and target density, maps the score to a dynamic gating coefficient, and fuses geometric cost, FastReID appearance cost, CLIP semantic cost, and dictionary retrieval cost under reliability constraints. Matching is finally solved using the Hungarian algorithm [45].

### 3.2. Quantitative Definitions of Semantic Noise and Semantic Collapse

To avoid treating semantic noise and semantic collapse as merely empirical descriptions, this paper provides computable definitions from the perspective of trajectory-detection candidate association. Let $F_{v}$ denote the frozen CLIP visual encoder and let $\left(I_{a},B_{a}\right)$ extract the target crop at bounding box $B_{a}$ from image $I_{a}$. After L2 normalization, the visual-semantic features $z_{a}$ and $z_{b}$ in $R^{512}$ of observations a and b are defined as follows:

(1) $$z_{a}=F_{v}(crop(I_{a},B_{a}))/||F_{v}(crop(I_{a},B_{a}))|{|}_{2},\;z_{b}=F_{v}(crop(I_{b},B_{b}))/||F_{v}(crop(I_{b},B_{b}))|{|}_{2},$$

The CLIP semantic similarity between them is defined as

(2) $$s(a,b)=z_{a}^{T}z_{b},$$

Let y(·) denote the identity label and c(·) denote the category label. The positive pair set P and the same-class negative pair set Nc are defined as

(3) $$P=\{(a,b)|y_{a}=y_{b},a\neq b\},$$

(4) $$N_{c}=\{(a,b)|y_{a}\neq y_{b},c_{a}=c_{b},(a,b)\in G\},$$

where G is the candidate-association gating set obtained from spatial overlap or motion-prediction constraints. The operator denotes intersection over union when spatial overlap is used. Pairs in P correspond to observations of the same identity at different moments, whereas pairs in $N_{c}$ correspond to candidate observations with the same category but different identities. Based on these sets, the semantic discriminative margin is defined as

(5) $$\Delta_{sem}=E_{(a,b)\in P}[s(a,b)]-E_{(a,b)\in N_{c}}[s(a,b)],$$

A larger Δsem indicates that CLIP semantic features can better distinguish the same identity from different identities within the same class. When Δsem approaches zero or becomes negative, different same-class identities are excessively close in the CLIP feature space, indicating weak instance-level discriminability.

For trajectory i, consider a frame t for which a ground-truth match is available. Let $j_{i,t}^{+}$ denote the true matched detection and let $N_{i,t}^{-}$ contain the incorrect same-class candidate detections that pass the candidate gate. The L2-normalized historical semantic prototype before frame t is defined as

(6) $${\bar{z}}_{i,t-1}=\frac{\sum_{\tau \in H_{i}^{t-1}}z_{i,\tau }}{{\left\|\sum_{\tau \in H_{i}^{t-1}}z_{i,\tau }\right\|}_{2}+\epsilon },$$

In Equation (6), $H_{i,t-1}$ denotes the set of valid observation frames associated with trajectory i before frame t. For each historical frame τ in $H_{i,t-1}$, $z_{i,\tau }$ in $R^{512}$ denotes the L2-normalized CLIP visual-semantic feature extracted from trajectory i’s observation at frame τ. The scalar ε > 0 is a small numerical constant used to prevent division by zero when the averaged feature vector has negligible norm. The numerator averages the historical feature vectors and the denominator normalizes this average; therefore, $\bar{z_{i,t-1}}$ is a unit-norm semantic prototype. The true-match similarity is

(7) $$s_{i,t}^{+}={\bar{z}}_{i,t-1}^{T}z_{j_{i,t}^{+},t},$$

Equation (8) uses the hardest negative candidate to quantify the most misleading same-class alternative. Here ${N_{i,t}}^{-}$ denotes the set of incorrect same-class detections that satisfy the candidate gate, and $z_{j,t}$ denotes the L2-normalized CLIP feature of candidate detection j in frame t. The maximum selects the candidate with the greatest similarity to the historical prototype, so $s_{i,t}^{-}$ represents the strongest competing semantic match; $s_{i,t}^{+}$ is the similarity to the true matched detection defined in Equation (7).

(8) $$s_{i,t}^{-}=\underset{j\in N_{i,t}^{-}}{\max}{\bar{z}}_{i,t-1}^{T}z_{j,t},$$

If si,t− ≥ si,t+, the incorrect same-class candidate detection is closer to the historical semantic prototype of the trajectory than the true matched detection in the CLIP semantic space. In this case, if the CLIP semantic cost is fused with a fixed weight, the semantic term may mislead data association. This situation is defined as a semantic noise event.

Let A denote the set of all association samples (i,t) for which a ground-truth matched detection exists and at least one incorrect candidate of the same category passes the candidate gate. In Equation (9), I(·) is the indicator function: it equals 1 when the condition inside the parentheses is true and 0 otherwise. Thus, SNR is the fraction of samples in A for which the hardest incorrect candidate is at least as similar as the true match, as measured by $s_{i,t}^{-}$ ≥ $s_{i,t}^{+}$. The Semantic Noise Rate (SNR) is defined as

(9) $$R_{SN}=\frac{1}{\left|A\right|}\sum_{(i,t)\in A}1\left(s_{i,t}^{-}\geq s_{i,t}^{+}\right),$$

A larger SNR means that CLIP semantic features more frequently rank an incorrect same-class candidate above the true match during candidate association.

To further measure the strength of semantic noise, the Semantic Noise Magnitude (SNM) is defined as

(10) $$M_{SN}=\frac{1}{\left|A\right|}\sum_{(i,t)\in A}\max\left(0,s_{i,t}^{-}-s_{i,t}^{+}\right),$$

To quantify semantic collapse, this paper defines the proportion of high-similarity pairs among same-class different-identity targets, namely the Semantic Collapse Index (SCI):

(11) $$C_{SC}(\tau )=\frac{1}{\left|N_{c}\right|}\sum_{(a,b)\in N_{c}}1\left(s(a,b)>\tau \right),$$

In the experiments, τ = 0.80 is used by default. $\Delta_{sem}$, SNR, SNM, and ${SCI}_{\tau }$ all depend on ground-truth identity labels. They are used only for offline analysis of CLIP semantic reliability in instance-level association, and do not participate in online tracking.

### 3.3. Candidate-Pair-Level Reliability-Aware Semantic Gating

CLIP features tend to represent category-level semantics. In dense same-class target scenarios, incorrect candidate detections may obtain semantic similarities close to, or even higher than, the true target. Since true identity labels cannot be directly observed during online tracking, semantic noise events cannot be explicitly identified. Therefore, this paper introduces a candidate-pair-level reliability estimation mechanism. Detection quality, spatial visibility, motion consistency, and target density are used as observable proxy variables to estimate the reliability of semantic cues in the association process.

For the candidate association pair formed by trajectory i and detection box j in frame t, the reliability vector is defined as

(12) $$r_{ij,t}={\left[\begin{array}{ccc} s_{j,t} & ,v_{j,t} & ,m_{ij,t} \end{array}\right]}^{⊤},$$

where sj,t ∈ [0, 1] denotes the normalized detection confidence of detection j, vj,t ∈ [0, 1] denotes its spatial visibility, and mij,t ∈ [0, 1] denotes the motion consistency between trajectory i and detection j.

The spatial visibility is computed from the overlap between the current detection and other detections in the same frame:

(13) $$o_{j,t}=\min\left(1,\sum_{k\neq j}\frac{\left|B_{j,t}\cap B_{k,t}\right|}{|B_{j,t}|+\varepsilon }\right),\;v_{j,t}=1-o_{j,t},$$

(14) $$d_{M}^{2}(hatx_{i,t},x_{j,t})=(x_{j,t}-hatx_{i,t}{)}^{T}S_{i,t}^{-1}(x_{j,t}-hatx_{i,t}),$$

The motion consistency is defined based on the Mahalanobis distance between the predicted state of trajectory i and the observation state of detection j:

(15) $$m_{ij,t}=\exp\left(-\frac{d_{M}^{2}\left({\hat{X}}_{i,t},X_{j,t}\right)}{\lambda_{m}}\right),$$

Equation (13) defines spatial visibility, Equation (14) gives the expanded Mahalanobis distance, and Equation (15) maps that distance to motion consistency. Here, $\hat{x_{i,t}}$ and $x_{j,t}$ are four-dimensional observation vectors ${\left[c_{x},c_{y},w,h\right]}^{T}$ representing the predicted trajectory state and the current detection, respectively. $S_{i,t}$ in $R^{4\times 4}$ is the innovation covariance matrix, and ${S_{i,t}}^{-1}$ is its inverse. The parameter $\lambda_{m}$ = 9.49 is the 95% chi-square scale for the four-dimensional motion gate. Equations (16)–(19) then define the reliability score, density terms, and dynamic semantic gate.

The basic reliability score of the candidate pair is defined as

(16) $$\rho_{ij,t}=\sigma (\beta^{T}r_{ij,t}+b),$$

where $(u)=\frac{1}{1+(-u)}$ is the sigmoid function, $\beta ={\left[0.40,\;0.35,\;0.25\right]}^{T}$ weights detection confidence, spatial visibility, and motion consistency, respectively, and b = −0.35 is the reliability-score bias.

To further consider scene crowdedness, the target density of frame t is defined as

(17) $$d_{t}=\min\left(\frac{N_{t}}{N_{\max}},1\right),$$

where N_t_ is the number of detections in frame t, and N_max_ is the normalization upper bound for the number of targets. Finally, the dynamic semantic gating coefficient is computed as

(18) $${\bar{s}}_{t}=\frac{1}{N_{t}}\sum_{j=1}^{N_{t}}s_{j,t},$$

where $\gamma_{c}=\;0.70,\gamma_{d}=\;0.70,\tau_{den}=\;0.30,\alpha_{min}=\;0.05,\alpha_{max}=\;1.00,\;and\;N_{max}=\;100$. The clipping operator is explicitly defined as (x, a, b) ={{x, a}, b}. The values were fixed on the parameter-selection split as manual starting values: β assigns the largest weight to detection confidence, while $\gamma_{c}$, $\gamma_{d}$, and $\tau_{den}$ regulate semantic terms when confidence or density becomes unreliable. $N_{max}$ is a normalization cap for per-frame target density, not a learned estimate of dataset size. The coupled gamma grid in Section 4.5 assesses only $\gamma_{c}$ and $\gamma_{d}$; a complete independent sensitivity study of all parameters remains work for the future.

(19) $$\alpha_{ij,t}=\mathrm{clip}(\rho_{ij,t}[1+\gamma_{c}({\bar{s}}_{t}-0.5)][1-\gamma_{d}\max(0,d_{t}-\tau_{den})],\alpha_{min},\alpha_{max}),$$

### 3.4. Retrieval-Augmented Trajectory Semantic Memory

This paper constructs a semantic dictionary and trajectory-level semantic memory. The dictionary contains a general category layer and two domain-related prompt layers, and the exact prompt templates are documented in Appendix B. In the current experiments, the prompt ensemble is used as an auxiliary retrieval reference, rather than as a complete industrial knowledge base.

Let the image of frame t be It, and let the detection box be Bj,t. The target region is cropped and fed into the CLIP visual encoder [16] to obtain the normalized semantic feature:

(20) $$z_{j,t}=\frac{F_{v}(crop(I_{t},B_{j,t}))}{\|F_{v}(crop(I_{t},B_{j,t})){\|}_{2}},$$

For an established trajectory i, its historical CLIP visual semantic prototype $z_{b}ar_{i,t}$ is maintained. When trajectory i is successfully matched with detection j, the trajectory semantic prototype is updated using the exponential moving average with $\eta_{z}$ = 0.10:

(21) $${\bar{z}}_{i,t}=\frac{(1-\eta_{z}){\bar{z}}_{i,t-1}+\eta_{z}z_{j,t}}{\|(1-\eta_{z}){\bar{z}}_{i,t-1}+\eta_{z}z_{j,t}{\|}_{2}},$$

Let pk be the prompt corresponding to the k-th dictionary entry. Its text semantic prototype is defined as

(22) $$e_{k}=\frac{F_{t}(p_{k})}{{\left\|F_{t}(p_{k})\right\|}_{2}},$$

where Ft(·) denotes the CLIP text encoder. The dictionary semantic prototype matrix is denoted as

(23) $$E={\left[e_{1},e_{2},\ldots ,e_{K}\right]}^{T},$$

For detection target j, the response distribution between its visual semantic feature and the dictionary text prototypes is defined as

(24) $$q_{j,t}=\mathrm{Softmax}\left(\frac{Ez_{j,t}}{\lambda_{q}}\right),$$

where $\lambda_{q}$ = 0.07 is the temperature coefficient. In practice, the dense dictionary matrix is stored in a FAISS IndexFlatIP index; only the top-k = 5 inner-product neighbors are retrieved for each detection, and the softmax is applied to this retrieved candidate set, rather than to the entire dictionary.

(25) $${\bar{q}}_{i,t}=(1-\eta_{q}){\bar{q}}_{i,t-1}+\eta_{q}q_{j,t},$$

When trajectory i is successfully matched with detection j, the dictionary memory is updated with $\eta_{q}$ = 0.10. This trajectory-level memory smooths single-frame semantic-response fluctuations and provides a stable auxiliary reference during occlusion recovery and appearance degradation.

### 3.5. Reliability-Constrained Multi-Cue Data Association

The core of online MOT is to establish reliable one-to-one correspondences between current-frame detections and historical trajectory states. Let the active trajectory set in frame t be

(26) $$T_{t-1}=\{\tau_{i}{\}}_{i=1}^{M},$$

and the detection set be

(27) $$D_{t}={\left\{d_{j}=(B_{j,t},s_{j,t},c_{j,t})\right\}}_{j=1}^{N_{t}},$$

For trajectory τi, Kalman filtering [44] is first used to predict its state and bounding box in the current frame, denoted as x^^^i,t and B^^^i,t, respectively. To reduce computational burden and false association risk caused by invalid candidate matches, a candidate gating mechanism is introduced before cost calculation. Only trajectory-detection pairs satisfying spatial overlap or motion constraints are retained:

(28) $$G_{t}=\left\{(i,j)|\mathrm{IoU}({\hat{B}}_{i,t},B_{j,t})\geq \theta_{iou\;}or{\;d}_{M}({\hat{x}}_{i,t},x_{j,t})\leq \theta_{m}\right\},$$

For candidate pair (i,j) ∈ Gt, four types of cues are integrated to construct the association cost: geometric motion, appearance representation, CLIP visual semantics, and dictionary retrieval semantics. The geometric cost is defined as

(29) $$C_{ij,t}^{g}=1-\mathrm{IoU}({\hat{B}}_{i,t},B_{j,t}),$$

The appearance cost is defined as

(30) $$C_{ij,t}^{a}=1-\frac{{\bar{f}}_{i,t-1}^{T}f_{j,t}}{\|{\bar{f}}_{i,t-1}{\|}_{2}\|f_{j,t}{\|}_{2}+\varepsilon },$$

The CLIP visual semantic cost is defined as

(31) $$C_{ij,t}^{s}=1-{\bar{z}}_{i,t-1}^{T}z_{j,t},$$

The dictionary retrieval cost is defined as

(32) $$C_{ij,t}^{d}=1-\frac{{\bar{q}}_{i,t-1}^{T}q_{j,t}}{\|{\bar{q}}_{i,t-1}{\|}_{2}\|q_{j,t}{\|}_{2}+\varepsilon },$$

Considering that CLIP visual semantics and dictionary retrieval semantics may suffer from semantic collapse in dense same-class target scenarios, this paper does not directly fuse semantic terms with fixed weights. Instead, the dynamic gating coefficient is introduced to regulate semantic-related costs in a reliability-aware manner. The final total cost of the candidate pair is defined as

(33) $$C_{ij,t}=w_{g}C_{ij,t}^{g}+w_{a}C_{ij,t}^{a}+\alpha_{ij,t}(w_{s}C_{ij,t}^{s}+w_{d}C_{ij,t}^{d}),$$

In this paper, $w_{g}=\;0.45,w_{a}=\;0.30,w_{s}=\;0.15,\;and\;w_{d}=\;0.08$. The FastReID appearance feature $f_{j,t}$ is a 2048-dimensional descriptor and is updated by the exponential moving average in the same manner as the semantic prototype. $C_{t}$ = [ $C_{ij,t}$ ] is the candidate-pair cost matrix. The Hungarian algorithm [45] solves the one-to-one assignment in Equation (34).

(34) $$\Pi_{t}^{*}=\arg\underset{\Pi_{t}}{\min}\sum_{(i,j)\in \Pi_{t}}C_{ij,t},$$

### 3.6. Online Algorithm Procedure

Algorithm 1 summarizes the reliability-aware semantic gating and retrieval-augmented association procedure. It accepts the current frame image, the current detection set, the historical trajectory set, and the semantic dictionary, and returns the updated trajectory set. Its core steps are detection acquisition, Kalman prediction, FastReID appearance extraction, CLIP feature extraction, FAISS top-k dictionary retrieval, candidate-pair reliability estimation, reliability-aware multi-cue cost construction, Hungarian matching, and trajectory-memory updating. For the reported MOT17 experiments, the supplied FRCNN detection set is used in place of detector inference.
**Algorithm 1:** Reliability-Aware Semantic Gating and Retrieval-Augmented MOT**Input:**$\mathrm{Current}\;\mathrm{frame}\;\mathrm{image}\;(I_{t}),$ $\mathrm{detection}\;\mathrm{set}\;(D_{t}),$ 
$\mathrm{historical}\;\mathrm{trajectory}\;\mathrm{set}\;(T_{t-1}),$ 
dual-level semantic dictionary (E).
**Output:**$\mathrm{Updated}\;\mathrm{trajectory}\;\mathrm{set}\;(T_{t}).$1$\mathrm{Detect}\;\mathrm{objects}\;(I_{t})$ and obtain detection boxes, categories, and confidence scores.2$\mathrm{for}\;\mathrm{each}\;\mathrm{trajectory}\;(\tau_{i}\in T_{t-1})$ do3$\;\;\;\;\mathrm{Predict}\;\mathrm{the}\;\mathrm{state}\;({\hat{x}}_{i,t})$ $\mathrm{and}\;\mathrm{bounding}\;\mathrm{box}\;({\hat{B}}_{i,t})$ using Kalman filtering.4end for5$\mathrm{for}\;\mathrm{each}\;\mathrm{detection}\;\mathrm{box}\;(B_{j,t}\in D_{t})$ do6$\mathrm{Crop}\;\mathrm{the}\;\mathrm{target}\;\mathrm{image}\;\mathrm{patch};\;\mathrm{extract}\;\mathrm{FastReID}\;\mathrm{appearance}\;\mathrm{feature}\;f_{j,t}$ 
$\mathrm{and}\;\mathrm{CLIP}\;\mathrm{visual}\;\mathrm{feature}\;z_{j,t}.$
7$\mathrm{Search}\;\mathrm{the}\;\mathrm{FAISS}\;\mathrm{IndexFlatIP}\;\mathrm{dictionary},\;\mathrm{retrieve}\;\mathrm{top}-k=5\;\mathrm{prompts},\;\mathrm{and}\;\mathrm{compute}\;q_{j,t}.$8$\;\;\;\;\mathrm{Compute}\;\mathrm{the}\;\mathrm{spatial}\;\mathrm{visibility}\;(v_{j,t}).$9end for10$\mathrm{Compute}\;\mathrm{the}\;\mathrm{average}\;\mathrm{detection}\;\mathrm{confidence}\;({\bar{s}}_{t})$ 
$\mathrm{and}\;\mathrm{target}\;\mathrm{density}\;(d_{t}).$
11$\mathrm{Initialize}\;\mathrm{the}\;\mathrm{total}\;\cos t\;\mathrm{matrix}\;(C_{t}).$12for each trajectory–detection pair ((i,j)) do13$\;\;\;\;\mathrm{if}\;((i,j)\notin G_{t})$ then14$\;\;\;\;\;(C_{ij,t}\leftarrow +\infty ).$15       continue16      end if17$\;\;\;\;\mathrm{Compute}\;\mathrm{the}\;\mathrm{geometric}\;\cos t\;(C_{ij,t}^{g}).$18$\mathrm{Compute}\;\mathrm{the}\;\mathrm{FastReID}\;\mathrm{appearance}\;\cos t\;C_{ij,t}^{a}.$19$\;\;\;\;\mathrm{Compute}\;\mathrm{the}\;\mathrm{CLIP}\;\mathrm{semantic}\;\cos t\;(C_{ij,t}^{s}).$20$\mathrm{Compute}\;\mathrm{the}\;\mathrm{dictionary}\;\mathrm{retrieval}\;\cos t\;C_{ij,t}^{d}$ from the retrieved FAISS response.21$\;\;\;\;\mathrm{Construct}\;\mathrm{the}\;\mathrm{reliability}\;\mathrm{vector}\;(r_{ij,t}=[s_{j,t},v_{j,t},m_{ij,t}{]}^{⊤}).$22$\;\;\;\;\mathrm{Compute}\;\mathrm{the}\;\mathrm{basic}\;\mathrm{reliability}\;\mathrm{score}\;(\rho_{ij,t}).$23$\;\;\;\;\mathrm{Compute}\;\mathrm{the}\;\mathrm{semantic}\;\mathrm{gating}\;\mathrm{coefficient}\;(\alpha_{ij,t}).$24      Compute the final association cost:
$\;\;\;\;(C_{ij,t}=w_{g}C_{ij,t}^{g}+w_{a}C_{ij,t}^{a}+\alpha_{ij,t}(w_{s}C_{ij,t}^{s}+w_{d}C_{ij,t}^{d})).$25end for 26$\mathrm{Apply}\;\mathrm{the}\;\mathrm{Hungarian}\;\mathrm{algorithm}\;\mathrm{to}\;(C_{t})$ 
$\mathrm{and}\;\mathrm{obtain}\;\mathrm{the}\;\mathrm{optimal}\;\mathrm{matching}\;\mathrm{result}\;(\Pi_{t}^{*}).$
27$\mathrm{for}\;\mathrm{each}\;\mathrm{successfully}\;\mathrm{matched}\;\mathrm{pair}\;((i,j)\in \Pi_{t}^{*})$ do28Update the Kalman state, FastReID appearance prototype, CLIP semantic prototype, and dictionary semantic memory.29end for 30Process unmatched trajectories and unmatched detections, remove expired trajectories, and initialize new trajectories.31$\mathrm{return}\;(T_{t}).$

## 4. Results

### 4.1. Experimental Settings

To verify the effectiveness of the proposed method in standard surveillance scenarios and to preliminarily examine its transfer limitations in low-altitude industrial-like scenes, experiments are conducted on the MOT17 training set [1] and the VisDrone2019-MOT-val industrial proxy subset [19]. For MOT17, a sequence-level split is used: MOT17-02, MOT17-04, and MOT17-05 are used only for parameter selection, while MOT17-09, MOT17-10, MOT17-11, and MOT17-13 are used for the reported validation results. No frame from the reporting split is used to tune the semantic-gating thresholds.

The VisDrone2019-MOT-val industrial proxy subset is used to approximate several visual characteristics of industrial low-altitude scenes, including overhead viewpoints, dense small targets, frequent occlusion, and complex backgrounds. Sequences are selected when they contain low-altitude UAV viewpoints, continuous multi-target interaction, and a sufficient number of annotated pedestrian/vehicle trajectories; empty or near-static clips are excluded. This subset is not a complete factory-floor MOT benchmark, and is not used to claim final deployment performance in real industrial scenarios. Instead, it is used as a stress test that exposes recall loss and detector-domain mismatch.

The evaluation follows the standard MOTChallenge protocol [1]. MOTA, IDF1, IDSw, MT, ML, precision, recall, and FPS are reported. HOTA [46] and AssA are additionally computed with the official TrackEval implementation on the same four-sequence MOT17 reporting split for FullModel and CLIP+FAISS.For instance-level reliability analysis of CLIP semantic features, the paper reports offline semantic discriminative margin, SNR, SNM, and SCI. These metrics require ground-truth identities and are used only for analysis, not online tracking.

All reported MOT17 results use the MOT17-09/10/11/13 reporting split, the supplied FRCNN detections, and seed 42, unless otherwise stated. The exact FullModel configuration is $w_{g}=\;0.45,w_{a}=\;0.30,w_{s}=\;0.15,\;and\;w_{d}=\;0.08$, $\gamma_{c}=\;0.70,\gamma_{d}=\;0.70$, and $\tau_{den}=\;0.30$, as listed in Table 1. Experiments use an NVIDIA GeForce RTX 3090 GPU (NVIDIA Corporation, Santa Clara, CA, USA) with PyTorch 2.0. For the VisDrone proxy reproduction, the detector front end is Ultralytics YOLOv8s 8.0.20 with COCO-pretrained weights, a 640 × 640 input, confidence threshold 0.25, and NMS threshold 0.70. Tracker parameters are max_age = 30, min_hits = 3, and match_threshold = 0.50. FastReID uses a ResNet-50 backbone with 2048-dimensional descriptors. CLIP-ViT-B/32 uses a 224 × 224 input and 512-dimensional L2-normalized embeddings. FAISS [47] uses IndexFlatIP with normalized dictionary entries, top-k = 5 retrieval, and $\lambda_{q}$ = 0.07.

Parameter-selection note: the listed values were fixed before the reported evaluation, and are not presented as globally optimal. Section 4.5 reports a coupled $\gamma_{c}$/$\gamma_{d}$ grid, but a one-at-a-time or joint sensitivity analysis for all critical parameters has not been completed.

### 4.2. Comparison with Mainstream Methods

To ensure fair comparison, the main comparison experiments include only tracking methods that can be reproduced under the same detection results, the same MOT17 reporting split, and the same evaluation protocol. The reporting split reduces leakage between parameter-selection and evaluation sequences, but all reported MOT17 results still come from the training set, rather than the official test server. Consequently, residual overfitting to the MOT17 training distribution cannot be excluded. Vision-language or end-to-end tracking methods such as OVTrack, GLEE, and MOTRv2 use different default detectors, training strategies, input resolutions, and model scales from this paper. Directly citing their original results would lead to unfair comparison. Therefore, results under different detectors and training settings are not mixed into the main performance table.

Table 2 reports the main comparison results on the MOT17 reporting split under FRCNN detections. The proposed method achieves 46.41% MOTA, 40.00% IDF1, 34.542% HOTA, 26.686% AssA, 382 ID switches, and 442 fragmentations. Compared with OC-SORT, the full model reduces both identity switches and fragmentations, but it does not dominate CLIP+FAISS retrieval in aggregate accuracy. This result is therefore better interpreted as an identity-stability trade-off than as a uniform performance gain.

Results are averaged over MOT17-09, MOT17-10, MOT17-11, and MOT17-13. IDSw and Frag are summed over the four sequences. FullModel uses CLIP and FAISS retrieval with the proposed DSG reliability gating. HOTA and AssA are computed with the official TrackEval implementation on the same four sequences for FullModel and CLIP+FAISS; -- indicates that the corresponding baseline was not evaluated with TrackEval.

All methods in Table 2 are evaluated on the same MOT17 reporting split with the supplied FRCNN detections. The HOTA and AssA entries use official TrackEval on seed 42 for FullModel and CLIP+FAISS; the MOTA, IDF1, and IDSw values for these two configurations were also reproduced identically with seeds 42, 100, and 2024. These reporting-split results should not be interpreted as an official MOT17 test-server evaluation.

### 4.3. Analysis of CLIP Semantic Noise and Semantic Collapse

Since the proposed semantic noise metrics require ground-truth identity labels and candidate-pair construction, the analysis focuses on same-ID similarity, same-class different-ID similarity, semantic discriminative margin, SNR, SNM, and SCI. The results are shown in Table 3.

In the low-density subset, the average same-ID similarity is 0.855, while the same-class different-ID similarity is 0.796, resulting in a discriminative margin of 0.0590. In the high-density MOT17-04 sequence, the same-class different-ID similarity increases to 0.817, and the discriminative margin decreases to 0.0451. Meanwhile, SNR increases from 12.7% to 21.4%, SNM increases from 0.018 to 0.031, and SCI increases from 18.3% to 32.6%. These results show that as target density increases, different identities belonging to the same category become more clustered in the CLIP semantic space. Consequently, CLIP category-level features may weaken instance-level discrimination when they are directly used for data association.

### 4.4. Unified Module Ablation Study

To verify the contribution of DSG, the ablation uses the same CLIP feature and FAISS retrieval pipeline under the MOT17 reporting split with FRCNN detections. Table 4 compares fixed semantic weighting without DSG against the FullModel with DSG.

The exact configuration was rerun with seeds 42, 100, and 2024. All three runs produced identical four-sequence totals. FullModel reports IDSw 382.0 +/− 0.0, MOTA 46.41 +/− 0.00, and IDF1 40.00 +/− 0.00; CLIP+FAISS reports IDSw 558.0 +/− 0.0, MOTA 47.25 +/− 0.00, and IDF1 39.32 +/− 0.00. Because the paired differences are identical in all three runs, this zero-variance result is reported as deterministic multi-seed replication, not as evidence of stochastic significance; no conventional *p*-value claim is made.

Adding DSG reduces identity switches from 558 to 382 (a relative reduction of 31.5%) and fragmentations from 670 to 442 (34.0%), while changing MOTA by −0.84 percentage points and increasing IDF1 by 0.68 points. HOTA increases from 34.178 to 34.542 and AssA increases from 26.172 to 26.686 under the official TrackEval evaluation. This result shows that DSG primarily improves identity stability, rather than aggregate detection accuracy.

### 4.5. Gamma Sensitivity Analysis

To assess the sensitivity of the coupled DSG parameters, $\gamma_{c}$ and $\gamma_{d}$ are varied together on the MOT17 reporting split with FRCNN detections. The grid covers $\gamma_{c}$ = $\gamma_{d}$ in {0.42, 0.56, 0.70, 0.84, 0.98}. This experiment evaluates the coupled gamma setting only; it does not establish independent sensitivity for every hyperparameter.

The baseline setting $\gamma_{c}$ = $\gamma_{d}$ = 0.70 is used in the FullModel. The neighboring values in Table 5 provide a local robustness check for this coupled setting, rather than a complete parameter-optimization study.

The table below reports the gamma grid and the resulting MOTA, IDF1, IDSw, and Frag values.

Across the coupled $\gamma_{c}$/$\gamma_{d}$ grid in {0.42, 0.56, 0.70, 0.84, 0.98}, MOTA varies by 0.02 percentage points, IDF1 by 0.08 points, and total IDSw by two switches (380–382). These results indicate low sensitivity within this tested coupled range.

### 4.6. Cross-Scenario Transfer Analysis on an Industrial Proxy Subset

**Figure 2:**
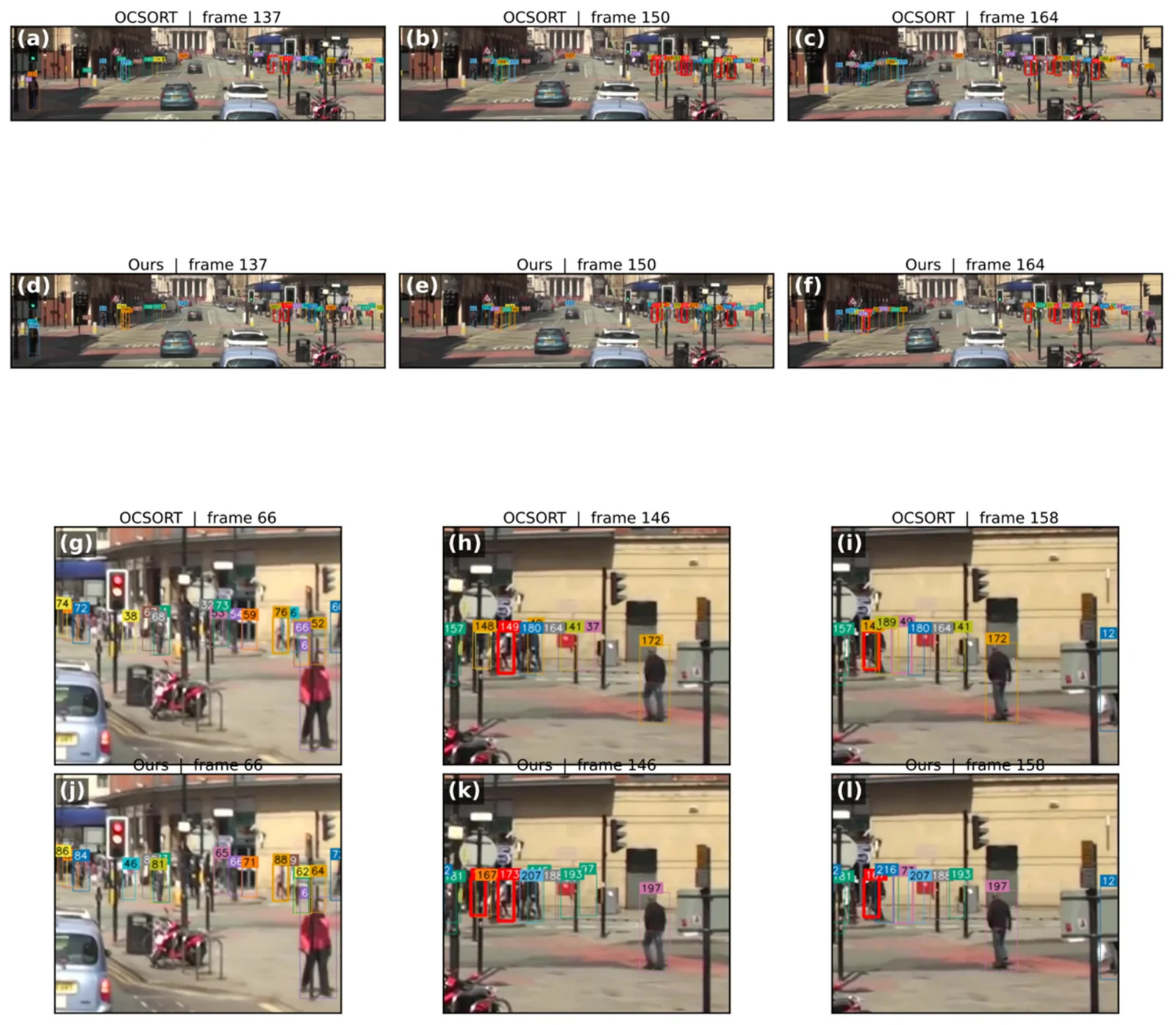
Qualitative tracking comparison under occlusion and homogeneous appearance in the industrial proxy subset. (**a**) OC-SORT tracking result on a frame with mild partial occlusion; (**b**) OC-SORT tracking result on a frame with severe inter-target occlusion; (**c**) OC-SORT tracking result on a frame with highly homogeneous target appearances; (**d**) OC-SORT tracking result on a frame with dense small targets; (**e**) OC-SORT tracking result on a frame with overhead viewpoint and scale variation; (**f**) OC-SORT tracking result on a frame with crossing target motions; (**g**–**l**) tracking results of the proposed method on the same frames as (**a**–**f**), respectively. Colored bounding boxes with numeric labels denote individual target trajectory identities; overlapping bounding boxes indicate targets in close spatial proximity or under partial occlusion, and are presented to visualize the instance-level association challenge rather than annotation errors.

To examine the transfer limitations and preliminary transfer potential of the proposed method, experiments are conducted on the VisDrone2019-MOT-val industrial proxy subset. This subset is used to approximate industrial low-altitude conditions, including overhead viewpoints, dense small targets, frequent occlusion, and complex backgrounds. It should be emphasized that this subset is not a complete factory-floor MOT benchmark. Qualitative tracking comparisons for occlusion and homogeneous-appearance scenarios are illustrated in Figure 2, where tracking outputs from OC-SORT and our proposed method are displayed side-by-side across matched video frames. Therefore, the results are used only as proxy evidence and as a way to expose domain-shift failure modes.

As shown in Table 6, the VisDrone proxy table reports MOTA, rather than HOTA. OC-SORT obtains −22.3% MOTA, 7.2% IDF1, 16.4% recall, and 45.2% precision. ByteTrack obtains −21.8% MOTA, 8.1% IDF1, 17.6% recall, and 46.5% precision. The proposed method without additional adaptation obtains −10.5% MOTA and 79.1% precision, but its recall is only 3.6% and its IDF1 is 5.3%. The 3.6% value is recall, not HOTA. The high precision therefore reflects a highly conservative tracker operating on very few positive predictions, not effective industrial MOT. This proxy experiment is retained as a failure-exposure and domain-shift stress test; it cannot support a claim of strong industrial performance.

### 4.7. Runtime Performance Analysis

Runtime efficiency is evaluated under two protocols. The tracker-level protocol excludes detector inference and is used only for comparison with reproduced baselines; the recorded tracker-level rate is 68.14 FPS. The end-to-end protocol includes YOLOv8s detection, target cropping, FastReID extraction, CLIP inference, FAISS retrieval, and data association, and runs at 21.7 FPS on the RTX 3090 platform. The current logs do not contain a component-level time breakdown, so the separate YOLOv8s, FastReID, CLIP, FAISS, and association shares cannot be reported without rerunning the profiler. The main optimization opportunities are batched crop inference, feature caching, selective CLIP extraction, and asynchronous or lightweight semantic inference.

### 4.8. Failure Analysis in Extremely Dense Scenes

**Figure 3:**
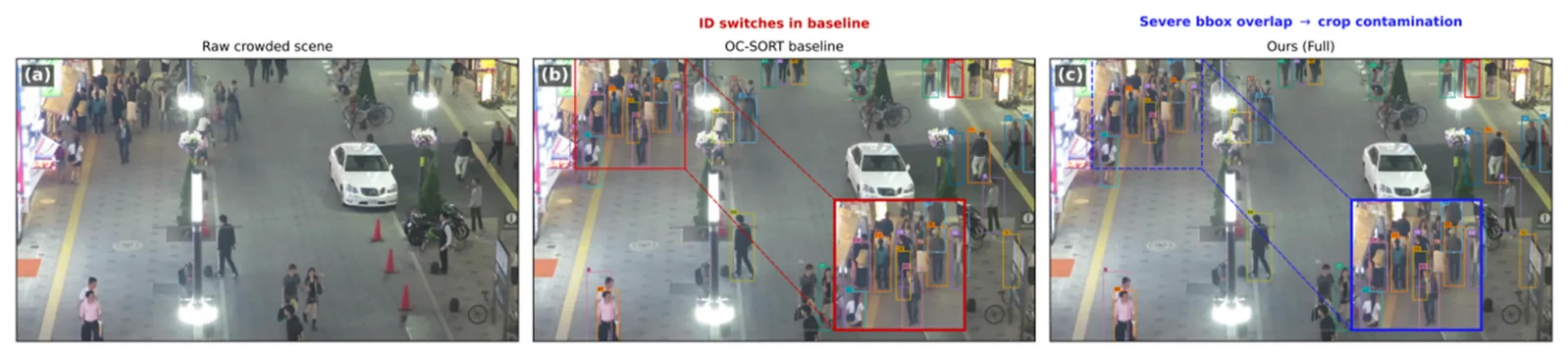
Failure-case analysis in extremely dense scenes. (**a**) Overview of an extremely dense crowded scene, showing the raw target distribution and severe bounding-box overlap; (**b**) baseline OC-SORT tracking results on the same scene, where the highlighted overlapping regions indicate identity-switch failures between adjacent same-class targets; (**c**) illustration of crop-contamination artefacts, where the highlighted target crop contains partial visual content of a neighboring target due to heavy bounding-box overlap. Colored bounding boxes with numeric identifiers represent assigned target trajectory identities; overlapping colored boxes in (**b**) mark identity-conflict regions, and the highlighted box in (**c**) indicates the contaminated target crop.

Although the proposed method improves identity preservation under normal and moderately crowded conditions, failure cases still occur in extremely dense scenes. When a large number of targets appear in a compact region, severe bounding-box overlap and inaccurate target crops may cause many cropped patches to contain neighboring targets or background regions. This reduces the instance-level discriminability of CLIP visual-semantic features. As a result, the dictionary response distributions of different same-class targets may become highly similar, and multiple candidate detections may satisfy comparable semantic and motion constraints. These problematic conditions are visualised in Figure 3, which highlights baseline identity-switch failures and crop-contamination artefacts caused by heavy bounding-box overlap.

In addition, long-term occlusion weakens the reliability of Kalman prediction. When a target disappears for many frames and reappears in a dense region, multiple candidate detections may satisfy similar motion and semantic constraints. Although DSG can reduce the contribution of unreliable semantic cues, it cannot fully compensate for severe detection misses, inaccurate bounding boxes, or long-term full occlusion. Therefore, the performance boundary of the proposed method is mainly determined by detection quality, crop reliability, and motion prediction stability in ultra-dense scenes.

## 5. Discussion

The experimental results show that category-level CLIP semantics must be reliability constrained when they are used for instance-level online association. In Table 4, fixed-weight CLIP+FAISS produces 558 identity switches and 670 fragmentations, whereas DSG reduces these values to 382 and 442, respectively. Official TrackEval results on the same four-sequence split give HOTA/AssA values of 34.178/26.172 for CLIP+FAISS and 34.542/26.686 for FullModel. Exact reruns with seeds 42, 100, and 2024 produced identical IDSw, MOTA, and IDF1 totals for each configuration; the resulting zero standard deviations indicate deterministic replication, not evidence of stochastic significance. This evidence supports an identity-stability effect; it does not establish a uniform gain in aggregate tracking accuracy.

The proposed dynamic semantic gating mechanism addresses this problem by estimating the reliability of semantic cues at the candidate-pair level. When detection confidence is high, occlusion is weak, motion prediction is consistent, and target density is low, semantic cues can participate more strongly in association. When the association state becomes unreliable, semantic costs are suppressed to avoid misleading matching. This design turns semantic features from fixed association cues into reliability-constrained auxiliary signals.

However, the proposed method has important limitations. First, the MOT17 result is a trade-off: the FullModel reduces identity switches relative to fixed-weight CLIP+FAISS, but its MOTA is 0.84 points lower. Second, the industrial evaluation uses a VisDrone-derived proxy subset, rather than a real factory-floor MOT benchmark; its 3.6% recall and 5.3% IDF1 show that the current system is not effective under this domain shift. Third, HOTA and AssA are reported for seed 42 only, while the exact three-seed reruns cover IDSw, MOTA, and IDF1 and yield zero sample standard deviation. This zero-variance outcome is a deterministic replication result, and does not justify a conventional *p*-value or stochastic-significance claim. Fourth, a complete independent sensitivity analysis for all parameters and a component-level end-to-end latency profile are not documented. Fifth, all MOT17 results use a reporting split from the training set, rather than the official test server, so residual overfitting risk remains. Additional boundary-case failure modes are further elaborated in Appendix A. These limitations define the scope of the current claims and motivate the future experiments below.

## 6. Conclusions

This paper investigates the reliability of CLIP semantic cues in online multi-object tracking and proposes a reliability-aware semantic gating and retrieval-augmented association framework. The main motivation is that CLIP features provide category-level semantic priors, but may introduce instance-level confusion when multiple same-class targets appear densely. To analyze this problem, a semantic discriminative margin, semantic noise rate, and semantic collapse index are introduced, to quantify the semantic ambiguity of CLIP features in trajectory-detection candidate association.

On the MOT17 reporting split, fixed-weight CLIP+FAISS obtains 47.25% MOTA, 39.32% IDF1, 34.178% HOTA, 26.172% AssA, 558 identity switches, and 670 fragmentations, whereas the FullModel obtains 46.41% MOTA, 40.00% IDF1, 34.542% HOTA, 26.686% AssA, 382 identity switches, and 442 fragmentations. DSG therefore reduces identity switches by 31.5% and fragmentations by 34.0%, with a 0.84-point MOTA trade-off and a 0.68-point IDF1 gain. The result supports the narrower claim that DSG improves identity stability under this protocol.

On the MOT17 reporting split with FRCNN detections, the proposed method achieves 46.41% MOTA, 40.00% IDF1, 382 ID switches, and 442 fragmentations. Compared with CLIP+FAISS retrieval, the full model is lower by 0.84 percentage points in MOTA and higher by 0.68 points in IDF1, while also reducing identity switches by 176 and fragmentations by 228, relative to the fixed-weight CLIP+FAISS baseline. The result should therefore be interpreted as a stability gain, rather than a uniform performance win.

Future work will focus on five directions. First, lightweight CLIP inference, feature caching, batch crop processing, and selective semantic extraction will be explored to reduce end-to-end overhead. Second, the prompt templates listed in Appendix B will be evaluated through controlled prompt ablations. Third, broader independent sensitivity studies, confidence intervals where nonzero variance is available, and statistically appropriate tests on independently varying runs will be pursued; the present three-seed result is reported only as deterministic replication. Fourth, official MOT17 test-server evaluation and additional UAV and industrial datasets will be used to reduce training-set evaluation bias. Fifth, a real industrial MOT dataset covering production lines, warehouses, conveyor facilities, and low-altitude inspection scenes will be constructed to evaluate practical deployment.

## Figures and Tables

**Table 1:** Hyperparameter settings of the proposed method.

Parameter	Value	Description
$w_{g}$	0.45	Base weight of geometric cost
$w_{a}$	0.30	Appearance cost weight
$w_{s}$	0.15	Base weight of CLIP visual semantic cost
$w_{d}$	0.08	Base weight of dictionary retrieval cost
β	[0.40, 0.35, 0.25]	Weights of detection confidence, spatial visibility, and motion consistency
$\tau_{den}$	0.30	Density threshold in Equation (19) selected on the parameter split
$\gamma_{d}$	0.70	Density suppression coefficient in Equation (19)
k	5	Number of retrieved nearest dictionary entries
match_threshold	0.50	Matching threshold of data association
max_age	30	Maximum unmatched frames for trajectory retention
min_hits	3	Minimum detection hits for trajectory confirmation
$N_{max}$	100	Upper bound of target number normalization
CLIP input size	224 × 224	Input resolution of CLIP visual encoder
Detector input size	640 × 640	Input resolution of YOLOv8s detector
$\gamma_{c}$	0.70	Detection-confidence modulation coefficient in Equation (19)
$\alpha_{min}$	0.05	Lower bound of the semantic gating coefficient
$\alpha_{max}$	1.00	Upper bound of the semantic gating coefficient
b	−0.35	Bias term of the reliability sigmoid in Equation (16)
$\lambda_{m}$	9.49	Mahalanobis scale for motion consistency in Equation (15)
$\eta_{z}$	0.10	EMA update rate for CLIP trajectory prototype
$\lambda_{q}$	0.07	Temperature for dictionary-response softmax
$\eta_{q}$	0.10	EMA update rate for dictionary semantic memory
YOLOv8s conf/NMS IoU	0.25/0.70	Detection confidence and non-maximum suppression thresholds
FastReID feature dimension	2048	ResNet-50 appearance descriptor dimension
CLIP feature dimension	512	ViT-B/32 visual-semantic descriptor dimension

**Table 2:** Main comparison on the MOT17 reporting split with FRCNN detections.

Method	MOTA (%)	IDF1 (%)	HOTA (%)	AssA (%)	IDSw (Total)	Frag (Total)
ByteTrack	39.64	49.85	--	--	810	297
OC-SORT	35.50	48.01	--	--	930	316
CLIP + FAISS	47.25	39.32	34.178	26.172	558	670
FullModel (ours)	46.41	40.00	34.542	26.686	382	442

**Table 3:** Quantitative analysis of CLIP semantic noise, including Semantic Noise Magnitude.

Scenario	Same-ID Similarity	Same-Class Diff-ID Similarity	Discriminative Margin	SNR	SNM	SCI (τ = 0.8)
Low-density subset	0.855	0.796	0.0590	12.7%	0.018	18.3%
High-density MOT17-04	0.862	0.817	0.0451	21.4%	0.031	32.6%

**Table 4:** Ablation study on the MOT17 reporting split with FRCNN detections.

Configuration	MOTA (%)	IDF1 (%)	HOTA (%)	AssA (%)	IDSw (Total)	Frag (Total)
CLIP + FAISS (fixed semantic weight, no DSG)	47.25	39.32	34.178	26.172	558	670
FullModel: CLIP + FAISS + DSG	46.41	40.00	34.542	26.686	382	442

**Table 5:** Gamma sensitivity of DSG on the MOT17 reporting split with FRCNN detections.

$\gamma_{c}$	$\gamma_{d}$	MOTA (%)	IDF1 (%)	IDSw (Total)	Frag (Total)
0.42	0.42	46.42	40.00	382	441
0.56	0.56	46.41	40.00	382	442
0.70	0.70 (baseline)	46.41	40.00	382	442
0.84	0.84	46.42	40.01	381	440
0.98	0.98	46.43	40.08	380	439

**Table 6:** Cross-scenario transfer results on the VisDrone2019-MOT-val industrial proxy subset.

Method	MOTA (%)	IDF1 (%)	IDSw	Precision (%)	Recall (%)
OC-SORT	−22.3	7.2	238	45.2	16.4
ByteTrack	−21.8	8.1	224	46.5	17.6
Proposed method	−10.5	5.3	146	79.1	3.6

## Data Availability

The original data presented in the study are openly available in MOTChallenge and VisDrone benchmark websites at https://motchallenge.net/ (accessed on 20 May 2025) and https://visdrone.org/ (accessed on 20 May 2025), respectively. The experimental results reported in this paper are based on these publicly available datasets.

## Abbreviations

The following abbreviations are used in this manuscript:

MOT	Multi-Object Tracking
CLIP	Contrastive Language-Image Pre-training
DSG	Dynamic Semantic Gating
FAISS	similarity-search library
MOTA	Multi-Object Tracking Accuracy
IDF1	Identity F1 Score
IDSw	Identity Switches
IoU	Intersection over Union
ReID	Re-identification
UAV	Unmanned Aerial Vehicle

## Appendix A. Failure Cases and Limitations

Although the proposed model improves identity stability in several settings, several boundary cases remain. First, in extremely dense scenarios, severe bounding-box overlap and inaccurate target crops may reduce the instance-level discriminability of CLIP visual-semantic features. Second, under long-term full occlusion, Kalman prediction based on the constant-velocity assumption may accumulate errors, making it difficult to recover the original identity when the target reappears. Third, under drastic illumination changes or severe crop misalignment, CLIP visual semantic features may exhibit short-term drift, affecting the stability of semantic association. Future work will improve the method through lightweight semantic encoders, more robust reliability estimation, and validation on real industrial MOT datasets.

## Appendix B. Semantic Dictionary Prompts

The following prompt templates are used by the retrieval-augmented semantic memory. {category} is replaced by the detector class label.

**Table A1:** General category layer.

Detail Level	Template
simple	A photo of a {category}
medium	A photo of a {category} in a typical setting
detailed	A high-quality photo of a {category} with clear features

**Table A2:** Industrial domain layer.

Detail Level	Template
simple	A {category} in a warehouse
medium	A photo of a {category} in an industrial warehouse setting
detailed	A photo of a {category} in an industrial warehouse setting, with typical warehouse lighting and background

**Table A3:** Warehouse domain layer.

Detail Level	Template
simple	A {category} in a warehouse
medium	A photo of a {category} in a warehouse environment
detailed	A photo of a {category} in a warehouse environment, with shelves, pallets, and industrial equipment in the background

The dictionary also uses the fixed three-prompt ensemble: a photo of a {category}, {category} in the scene, and high quality image of {category} when encoding a category name.
